# CD24 Expression and B Cell Maturation Shows a Novel Link With Energy Metabolism: Potential Implications for Patients With Myalgic Encephalomyelitis/Chronic Fatigue Syndrome

**DOI:** 10.3389/fimmu.2018.02421

**Published:** 2018-10-22

**Authors:** Fane F. K. Mensah, Christopher W. Armstrong, Venkat Reddy, Amolak S. Bansal, Saul Berkovitz, Maria J. Leandro, Geraldine Cambridge

**Affiliations:** ^1^Division of Medicine, Centre of Rheumatology Research, University College London, London, United Kingdom; ^2^Bio 21 Molecular Science and Biotechnology Institute, University of Melbourne, Melbourne, VIC, Australia; ^3^Department of Immunology, Epsom and St. Helier University Hospitals NHS Trust, Carshalton, United Kingdom; ^4^Chronic Fatigue Service, Royal London Hospital of Integrated Medicine, University College Hospitals NHS Trust, London, United Kingdom

**Keywords:** B cells, CD24, metabolism, ME/CFS, memory B cells, pAMPK

## Abstract

CD24 expression on pro-B cells plays a role in B cell selection and development in the bone marrow. We previously detected higher CD24 expression and frequency within IgD+ naïve and memory B cells in patients with Myalgic Encephalomyelitis/Chronic Fatigue Syndrome (ME/CFS) compared with age-matched healthy controls (HC). Here, we investigated the relationship between CD24 expression and B cell maturation. *In vitro* stimulation of isolated B cells in response to conventional agonists were used to follow the dynamics of CD24 positivity during proliferation and differentiation (or maturation). The relationship between CD24 expression to cycles of proliferation and metabolism in purified B cells from HC was also investigated using phospho-flow (phosphorylation of AMPK-pAMPK), 1proton nuclear magnetic resonance and Mitotracker Far-red (Mitochondrial mass-MM). *In vitro*, in the absence of stimulation, there was an increased percentage of CD24+ viable B cells in ME/CFS patients compared to HC (*p* < 0.05) following 5 days culture. Following stimulation with B cell agonists, percentage of CD24+B cells in both naïve and memory B cell populations decreased. *P* < 0.01). There was a negative relationship between percentage of CD24+B cells with MM (R^2^ = 0.76; *p* < 0.01), which was subsequently lost over sequential cycles of proliferation. There was a significant correlation between CD24 expression on B cells and the usage of glucose and secretion of lactate *in vitro*. Short term ligation of the B cell receptor with anti-IgM antibody significantly reduced the viability of CD24+ memory B cells compared to those cross-linked by anti-IgD or anti-IgG antibody. A clear difference was found between naïve and memory B cells with respect to CD24 expression and pAMPK, most notably a strong positive association in IgD+IgM+ memory B cells. *In vitro* findings confirmed dysregulation of CD24-expressing B cells from ME/CFS patients previously suggested by immunophenotype studies of B cells from peripheral blood. CD24-negative B cells underwent productive proliferation whereas CD24+ B cells were either unresponsive or susceptible to cell death upon BCR-engagement alone. We suggest that CD24 expression may reflect variations in energy metabolism on different B cell subsets.

## Introduction

CD24 is one of the earliest expressed proteins during human B cell maturation, being present at the late pro-B cell stages alongside surface markers such as CD21 and cytoplasmic μ heavy chains (1, 2). CD24 is a highly glycosylated protein which is glycosyl-phosphatidylinositol-anchored (GPA) but can be localized to lipid rafts on the plasma membrane of B cells (3). Its role was first described following the finding that cross-linking CD24 on immature pro- and pre-B cells B cells could block B cell development in a murine model by inducing apoptosis (4, 5). The dynamic regulation of CD24 on immature bone marrow B cells and its role in apoptosis has been confirmed in *in vitro* cell cultures of mouse and human cell lines and was thus suggested to be involved in determining the fate of B lymphoid progenitor cells (6–8). The selection process that results in apoptosis of many autoreactive B cells in the bone marrow is complex but involves both the specificity of the B cell receptor (BCR) and other signaling molecules, including CD24 (1, 9, 10). For example, transgenic mice overexpressing CD24 exhibit a loss of late pre- and immature B cells due to increased apoptosis (11). Cross-linking or engagement of CD24 may regulate BCR-mediated B cell selection in the bone marrow, consequently, the generation and emigration of transitional B cells to the periphery.

In the peripheral lymphoid system of humans, CD24 expression undergoes continuous fluctuations in expression throughout the lifespan of mature B cells until CD24 is lost when B cells differentiate into antibody-producing cells (12–14). Although the functional consequences of the changes in CD24 expression on mature naïve and memory B cells have been poorly studied in the human, Sanz and colleagues have described high expression patterns of CD24 in the majority of CD27+ B cells while the majority of CD27− B cells had low expression in healthy subjects. Isotype analysis within the CD27+ and CD27− B cell subsets revealed that IgM-only cells in both subsets are a distinctive population of CD24+B220-(CD45R) cells. On the contrary, IgG switched memory B cells were heterogeneous in the expression of CD24 and B220 (15). While previous studies focused on experiments crosslinking (or engaging) and overexpression of CD24 molecules in murine models, the functional consequences of the changes in CD24 expression on mature peripheral blood-derived naïve and memory B cells has been poorly studied in human health and disease.

We recently described significantly increased frequency and expression of CD24 on subsets of IgD+IgM+ B cells from patients suffering from Myalgic Encephalomyelitis/Chronic Fatigue Syndrome (ME/CFS) (16), a multisystem disorder characterized by fatigue, post-exertional malaise and cognitive impairment (17, 18). Although CD24 plays a well-described role in early B cell development in the bone marrow in mice and man, our novel finding of increased CD24 on B cells as a potential biomarker for ME/CFS patients prompted the investigation of its possible function throughout B cell maturation in the periphery. Here we investigated the *in vitro* behavior of CD24 following B cell-directed stimulation. We describe a potential role for CD24 in the generation and maintenance of B cell fate in IgM+ memory B cells likely mediated through a metabolic pathway involving phosphorylation of AMPK.

## Materials and methods

### Patients and healthy controls

Patients diagnosed with ME/CFS fulfilling the revised Canadian Consensus Criteria (CCC 2010; incorporating Canadian, CDC and Fukuda criteria) were selected for the study at 2 ME/CFS referral centers, namely the Royal London Hospital of Integrated Medicine, UCLH NHS Foundation Trust (under the care of Dr. S. Berkovitz) and St. Helier Hospital NHS Trust (under the care of Dr. A. Bansal). Nine ME/CFS patients (*6F, 3M; median age 33; range 22-52)* and 8 healthy controls (HC) (*5F, 3M*; median age *33; range 23–63*) were included. Length of history ranged from 4 to 21 years. All were Caucasian, time of blood taking, where 0 is no symptoms and 10 most severe) were 7.3 for fatigue, 4.3 for cognitive impairment and 4 for pain. Subjects were sourced from the same cohort of patients and controls described in detail previously (16). Informed consent was obtained and medical history (disease duration, the severity of symptoms and co-morbidities) was recorded for the purpose of the study. Patients with a confirmed history of autoimmune disease or receiving immunosuppression were excluded, as well as those who had a history of depression (HADS >17). Exclusion criteria for the control group was if a first or second degree relative of a ME/CFS patient. Healthy controls were recruited from hospital and academic staff and volunteers amongst friends of patients without evidence of ME/CFS on the basis of completed symptom questionnaires. This study has been approved by the NRES Committee London-City Road & Hampstead Research Ethics Committee (REC reference: 14/LO/0388).

### Cell cultures and stimulations

Whole blood from ME/CFS patients and healthy controls were used to isolate peripheral blood mononuclear cells (PBMCs) by centrifugation over Lymphoprep (Stemcell^TM^ Technologies, Vancouver BC). B cells were enriched by negative selection using the EasySep^TM^ Human B cell isolation kit (Stemcell^TM^ Technologies, Vancouver BC). B cells were then stained with the fluorescent dye Carboxyfluorescein succinimidyl ester (CFSE; Biolegend, San Diego CA) to follow proliferation. PBMCs or CFSE stained B cells (5 × 10^4^ per well in 96 well flat bottom plates) were cultured in the presence of T-dependent (TD)—anti-CD40 (LEAF™ Purified anti-human, Biolegend San Diego CA) anti-IgM [AffiniPure F(ab')_2_ Fragment Goat Anti-Human IgM, Fc5μ fragment specific, West Grove PA] and IL2 (Human, PeproTech EC Ltd, Rocky Hill NJ), or additional stimulation through Toll-like receptor 9 with CpG oligodeoxynucleotides (ODN 2006, Invivogen, San Diego CA) + anti-IgM and IL2) or with B cell activating factor (BAFF; R&D systems, Minneapolis MN), a B cell survival cytokine (19). After 5 days of culture in RPMI-1640 (Sigma-Aldrich, St Louis MO) supplemented with 10% fetal bovine serum (FBS; Labtech International, Heathfield UK), B cells were harvested for flow cytometry analysis and supernatants from cultures were collected and analyzed for immunoglobulins and sCD23 production.

### Flow cytometry

PBMCs or B cells were stained for 20 min with fluorescent conjugates of CD19-Alexa Fluor 700, CD38-PerCP.Cy5.5, CD39-FITC and CD73-PE (Biolegend, San Diego, CA) IgD-BV421, IgM-BV605 (BD Biosciences, San Jose, CA) CD27−APC and CD24-APC eFluor780 (eBioscience, San Diego, CA), and a viability marker (LIVE/DEAD^TM^ Fixable aqua dead cell stain, ThermoFisher Scientific, Waltham, MA). Cells were washed (centrifuged for 5 min 300 × g at room temperature) and resuspended in PBS and acquired within 24 h on a BD LSR Fortessa^TM^X-20. Compensation beads (BD, Biosciences, San Jose, CA) were used to optimize fluorescence compensation settings for multicolour flow cytometric analysis. A minimum of 100,000 events in the lymphocyte gate was collected. Naïve and Memory B cell subsets were defined as in our previous publication (16) based on the classification described in Ref (20). Representative plots of the classical B-cell subsets defined by IgD/CD27 and IgD/CD38 using this system are shown in Supplementary Figure 1.

### Staining of mitochondrial mass in B cells with mitotracker^TM^ red FM

Freshly isolated PBMCs or cultured B cells were incubated with 22 nM Mitotracker^TM^ Red FM (ThermoFisher Scientific, Waltham, MA) in preheated (37°C) RPMI medium 1,640 without serum for 30 min at 37°C. Cells were then washed (5 min 300 × g at RT) with RPMI, the pellet resuspended in PBS and stained for 20 min with CD19-Alexa Fluor 700, CD27−APC, CD24-APC eFluor780 and IgD-BV421 as indicated above.

### Relationship between B cell membrane molecules and glycolysis during *in vitro* stimulation: ^1^proton nuclear magnetic resonance (^1^H NMR)

Briefly, 5 × 10^5^ B cells/well from 6 Healthy donors were cultured in the presence of BAFF, anti-CD40 + (anti-IgM+IL2) and CpG + (anti-IgM+IL2) stimulation (as above) in 1.5 ml complete medium for up to 6 days in 24 wells culture plates with transwell inserts (Corning, New York, NY). 500 uL of culture supernatants were sampled at days 1 and 3 of which 200 uL was combined with 200 uL of ice-cold methanol-d4 (Sigma-Aldrich, St Louis MO), allowed to rest for 3 min, then 200 μL ice-cold deuterated chloroform-d (Sigma-Aldrich, St Louis MO) was added and mixed by vortexing. Samples were then centrifuged (13,000 rpm) at 4°C for 5 min to produce a biphasic mixture with a hydrophilic phase of water/deuterated methanol and lipophilic phase of deuterated chloroform. A 300 μL sample of the top hydrophilic layer was added to 300 uL of 200 mM sodium phosphate in D2O (pH 7) containing 2 mM DSS and 0.2% (w/v) sodium azide. 550 μL of supernatant was transferred to a 7-inch 5-mm 507-grade NMR tube and run on 700 MHz (Bruker Avance Neo 700) for NMR analysis. Metabolites in samples from cultures were analyzed as previously described by one of us (21). Flow cytometry was used to measure percentages of live B cells and changes in B cell phenotype markers (CD19, CD27, IgD, IgM, and CD24) and additional surface markers involved in energy pathways namely CD73, CD39, and CD38 at the same time points.

### Detection of phosphorylated adenosine monophosphate kinase (pAMPK) in B cells

Purified B cells were stained for 20 min with CD19-Alexa Fluor 700, CD27−APC and CD24-APC eFluor780 as indicated above. Stained cells were then fixed with Cytofix fixation buffer (BD Biosciences, San Jose, CA) for 10 min at 37°C, followed by a wash step (4 min 1,800 rpm at 4°C) cells were permeabilized with Phosphoflow Perm buffer (BD Biosciences, San Jose, CA) for 30 min at 4°C. After washing, cells were stained with Phospho-AMPK Alpha rabbit monoclonal antibody (Cell Signaling Technology, Danvers, Massachusetts) for 30 min at RT. Cells were then washed and stained with a fluorochrome-conjugated secondary antibody, Goat anti-Rabbit FITC (Vector Laboratories, Burlingame, CA) for 30 min. After the final wash step cells were fixed with 2% PFA and acquired on the flow cytometer directly.

### Measurement of soluble factors associated with B cells survival and differentiation

Commercial ELISA kit was used to measure serum soluble CD23 (sCD23) production (R & D systems Europe Ltd; Abingdon, UK). Following B cell activation, surface expression of CD27 induces cleavage of CD23 and can for that reason be used as a measurement of B cell turnover from naïve to memory B cells (22, 23). Relative sCD23 levels were used to confirm the differentiation of stimulated B cells from naïve to memory status. Human IgM and IgG total ELISA Ready-SET-Go!® (eBioscience, San Diego, CA) kits were used to measure antibody production in culture supernatants.

### Statistical analysis

Comparisons of levels of serum factors and B cell phenotype parameters between patients with ME/CFS patients and healthy controls and between B cell subsets were made using non-parametric tests (Mann-Whitney *U*-test), non-parametric multiple comparisons (One-way ANOVA) and linear regression (Pearson correlation coefficient) using Graph Pad Prism 6 (GraphPad, San Diego, USA) with significance level of 5% (*p* < 0.05 ^*^).

## Results

### CD24 expression during B cell maturation

**Figure 1:**
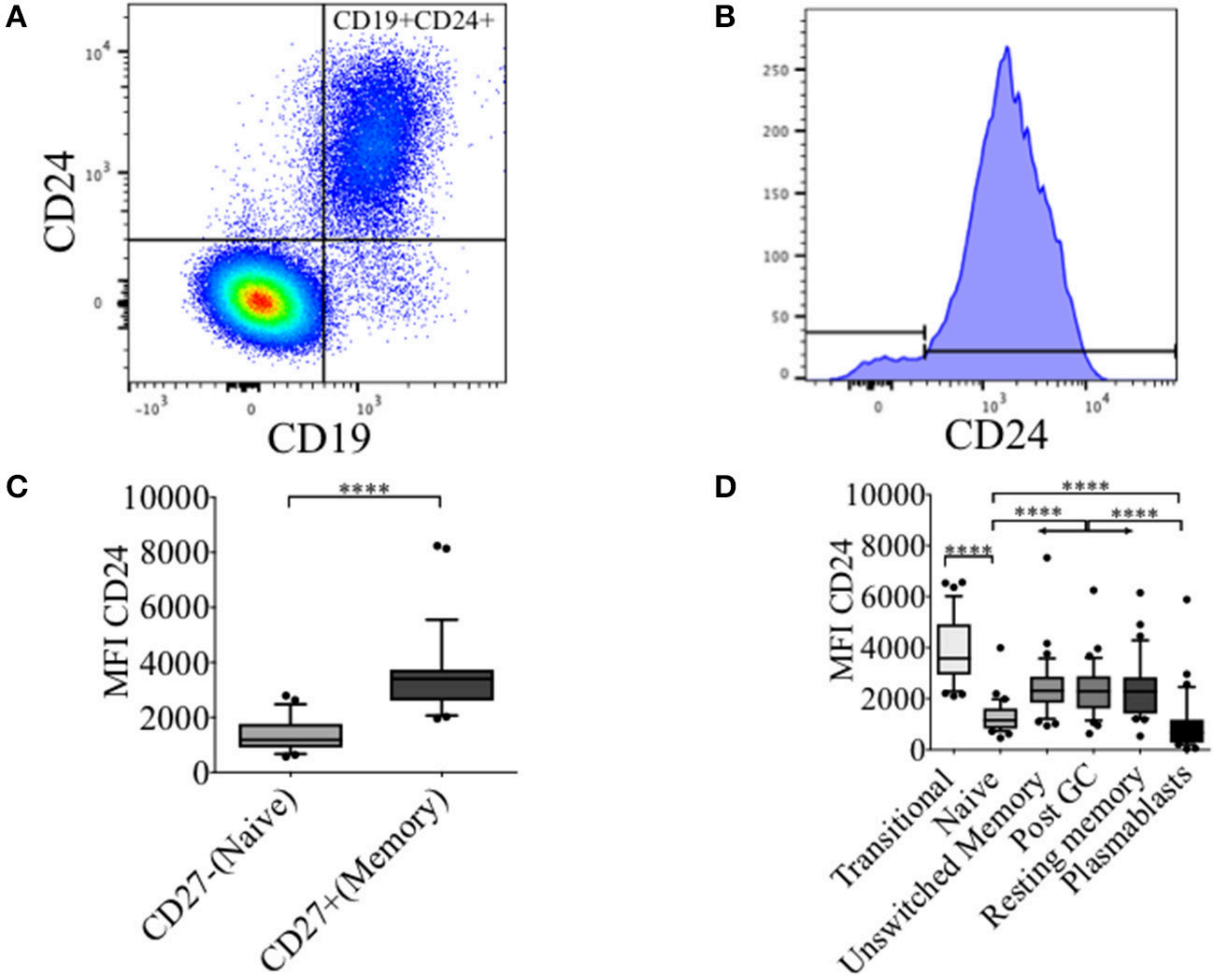
Dynamics of CD24 expression throughout B cell maturation. Representative flow cytometric dot plot showing the gating strategy for CD24+CD19+ B cells **(A)** and a histogram of CD24 expression within total B cells **(B)** from freshly isolated PBMCs of a healthy control. Box and whiskers plots show the expression of CD24 in CD27− and CD27+ B cells **(C)** and B cell subsets distinguished using the relative expression of IgD and CD38 **(D)**. Box and whiskers plots show median and 10th to 90th percentile in (HC; *N* = 32). ****p < 0.0001 (Mann-Whitney *U*-test).

In healthy controls (HC) we have previously described an increased frequency (%) of CD24 expressing un-switched-memory B cells (IgD+CD38-) compared to that of naïve B cells (IgD+CD38+), and that the percentage positive for CD24 subsequently decreased in post-germinal center (IgD-CD38+) resting memory (IgD-CD38-) and plasmablasts (IgD-CD38++) (16). In Figures 1A,B, the distribution of CD24+ B cells within total lymphocytes and CD19+ B cell populations are shown. The majority of B cells expressed CD24; non-B cells (CD19- cells) which were mainly T cells did not express CD24. As shown in Figure 1C, CD24 expression in CD27+ B cells was significantly higher than in CD27−B cells (*p* < 0.0001). We also determined levels of CD24 expression (MFI) on B cell subsets defined using the relative expression of IgD and CD38. CD24 expression of transitional B cells newly exited from the bone marrow through to antibody-producing plasmablasts is shown for CD19 gated B cells (Figure 1D) in HC. CD24 expression was found to be highest on transitional B cells (IgD+CD38++), declined in mature naïve B cells (*p* < 0.0001) with expression significantly increased in all 3 memory B cell populations (Unswitched memory, Post GC and Resting memory B cells respectively) compared to naïve B cells (*p* < 0.0001 One-way ANOVA). Plasmablasts showed the lowest expression of CD24 with a significant difference from naïve mature (*p* < 0.0001) and memory B cell subsets (*p* < 0.0001). Expression of CD24 thus changed significantly throughout B cell maturation.

### The effect of stimulation on %CD24+ B cells in PBMCs from healthy controls and ME/CFS patients

**Figure 2:**
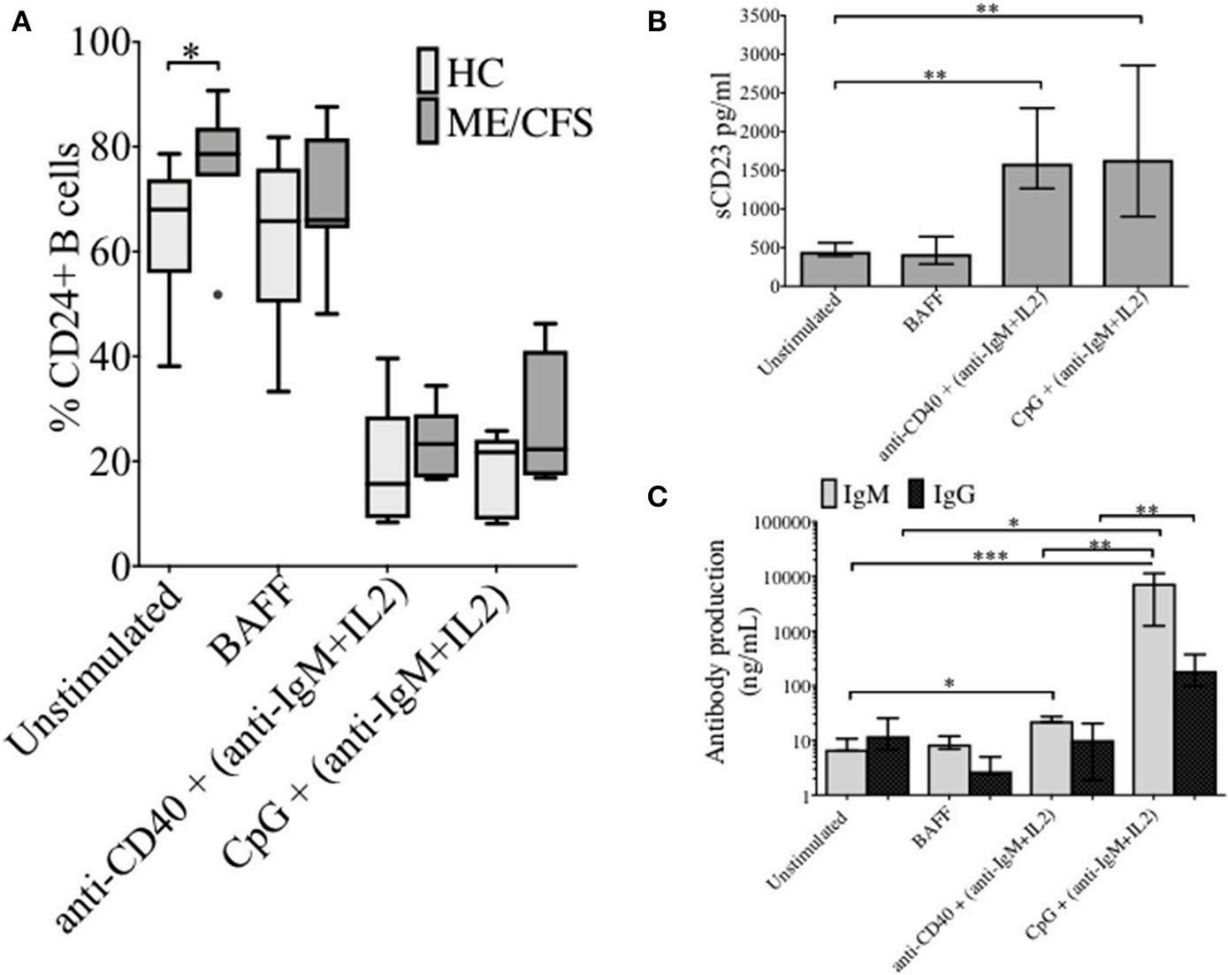
CD24+ B cells, soluble CD23 release and antibody production following 5 days culture of PBMCs with different stimuli. The frequency of CD24+ B cells in HC (*N* = 6) and ME/CFS patients (*N* = 9), after *in vitro* culture for 5 days is shown **(A)**. Box and whiskers show medians and 10th to 90th percentiles. Levels of sCD23 **(B)**, IgG and IgM antibody production **(C)** in the supernatants of cultures are shown as a measure of differentiation. Bars show median with interquartile range *p < 0.05, **p < 0.01, ***p < 0.001 (Mann-Whitney *U*-test).

The changes in %CD24+ B cells in PBMCs after *in vitro* stimulation with either anti-CD40 + (anti-IgM+IL2) or CpG + (anti-IgM+IL2) induced stimulation was followed. CpG stimulation utilized a synthetic oligonucleotide (CpG ODN-2006) that binds Toll-Like receptor 9, whereas stimulation with anti-CD40 antibody mimics T cell dependent (TD) stimulation. Both culture conditions were combined with the T cell proliferation cytokine IL2 and anti-IgM which cross-links the B cell receptor, and thus predominantly targets naïve and IgM memory B cells. PBMCs were also cultured in medium alone or in the presence of the pro-survival B cell cytokine BAFF. After 5 days of culture, cells were harvested and stained with a viability stain, along with CD19 and CD24 fluorescent-conjugated monoclonal antibodies. Culture supernatants were retained for measurements of sCD23. After 5 days, the frequency of CD24+ B cells (CD19+) in cultures incubated with different stimuli was compared between HC and ME/CFS patients (Figure 2A). Unstimulated and BAFF containing cultures maintained higher frequencies (%) of CD24+ B cells after 5 days of culture. The %CD24+ B cells steeply decreased after both anti-CD40 and CpG stimulation. A significant increase in the %CD24+B cells compared with HC was found in PBMCs from ME/CFS patients (*p* < 0.05) in unstimulated cultures. Following B cell activation, surface expression of CD27 is associated with cleavage of CD23 from the B cell. As shown in Figure 2B, a decrease in CD24 expression after stimulation was associated with an increase of sCD23 in culture supernatants, while unstimulated and PBMCs cultured with BAFF did not release CD23, confirming lack of *in vitro* differentiation. Post-germinal center and other memory B cells are negative for CD23 and the relative level of the soluble factor (sCD23) cleaved from the B cell surface during differentiation to memory status has been used by us and others as a surrogate measure of B cell activation/differentiation from the naïve (20, 22, 23). No differences were found between ME/CFS patients and HC in sCD23 levels (data not shown). IgM and IgG levels in the same culture supernatants are shown in Figure 2C, confirming differentiation to Ig production by naïve and memory B cells in CpG stimulated cultures, but not those stimulated under TD (anti-CD40) conditions. It has previously been shown that TD stimulation through CD40L induced proliferation of both naïve and memory B cells, but not differentiation to plasma cells, whereas CpG induced memory B cell differentiation resulted in immunoglobulin production following proliferation and differentiation to plasma cells (24).

### Frequency of CD24+ naïve and memory B cells over sequential proliferation cycles following TI stimulation

**Figure 3:**
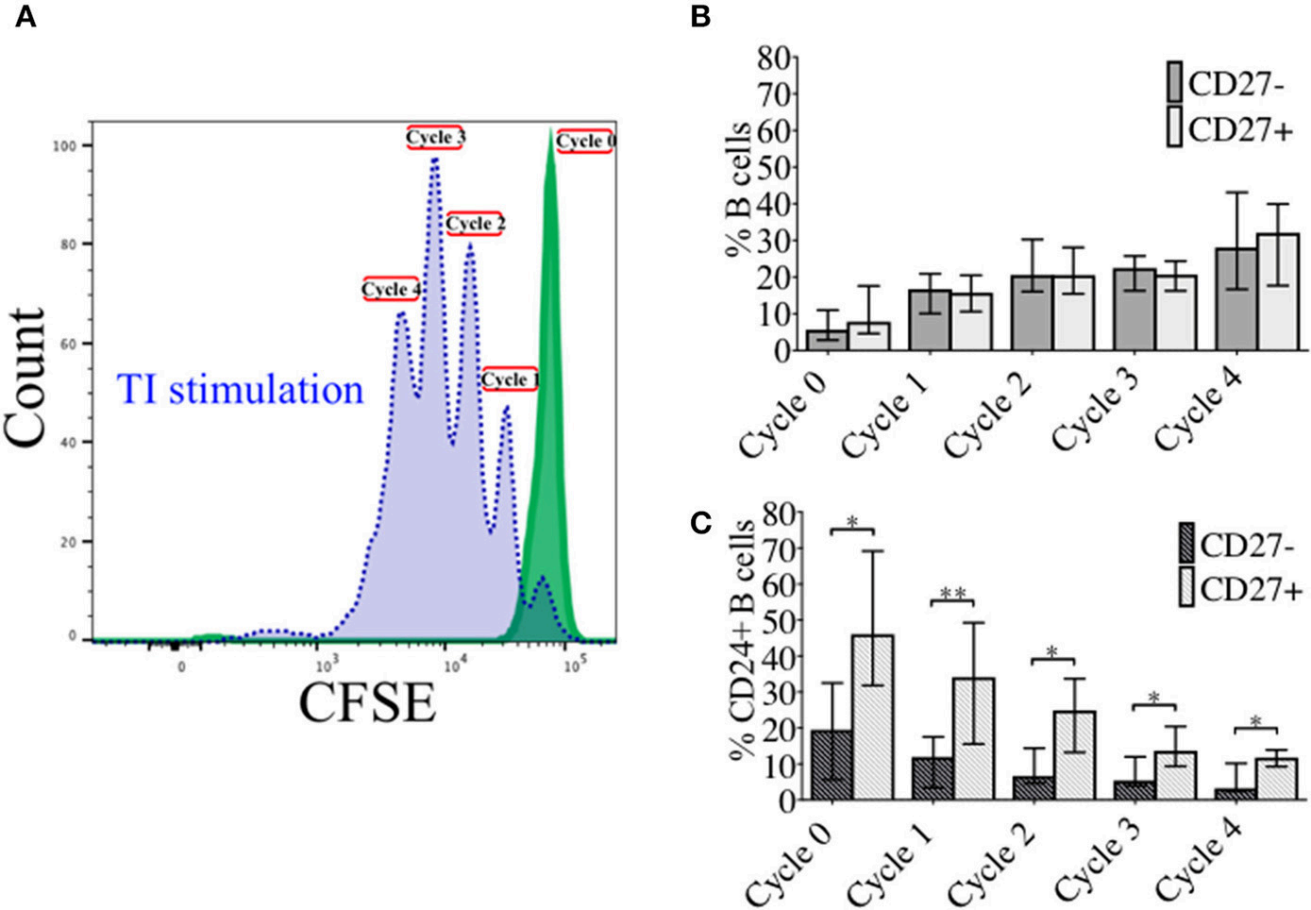
Percentage CD24+ B cells over sequential proliferation cycles in CD27− and CD27+ B cells. A representative plot of proliferation cycles from isolated B cells when incubated with BAFF (solid line; green) and CpG + (anti-IgM+IL2) stimulation (dotted line; blue) after 5 days of culture is shown **(A)**. The median ± interquartile range of percentage CD27− and CD27+ B cells in total B cells **(B)** and CD24+ B cells **(C)** over 5 sequential proliferation cycles are shown in median bars with interquartile range (HC; *N* = 8). *p < 0.05 and **p < 0.005 (Mann-Whitney *U*-test).

**Figure 4:**
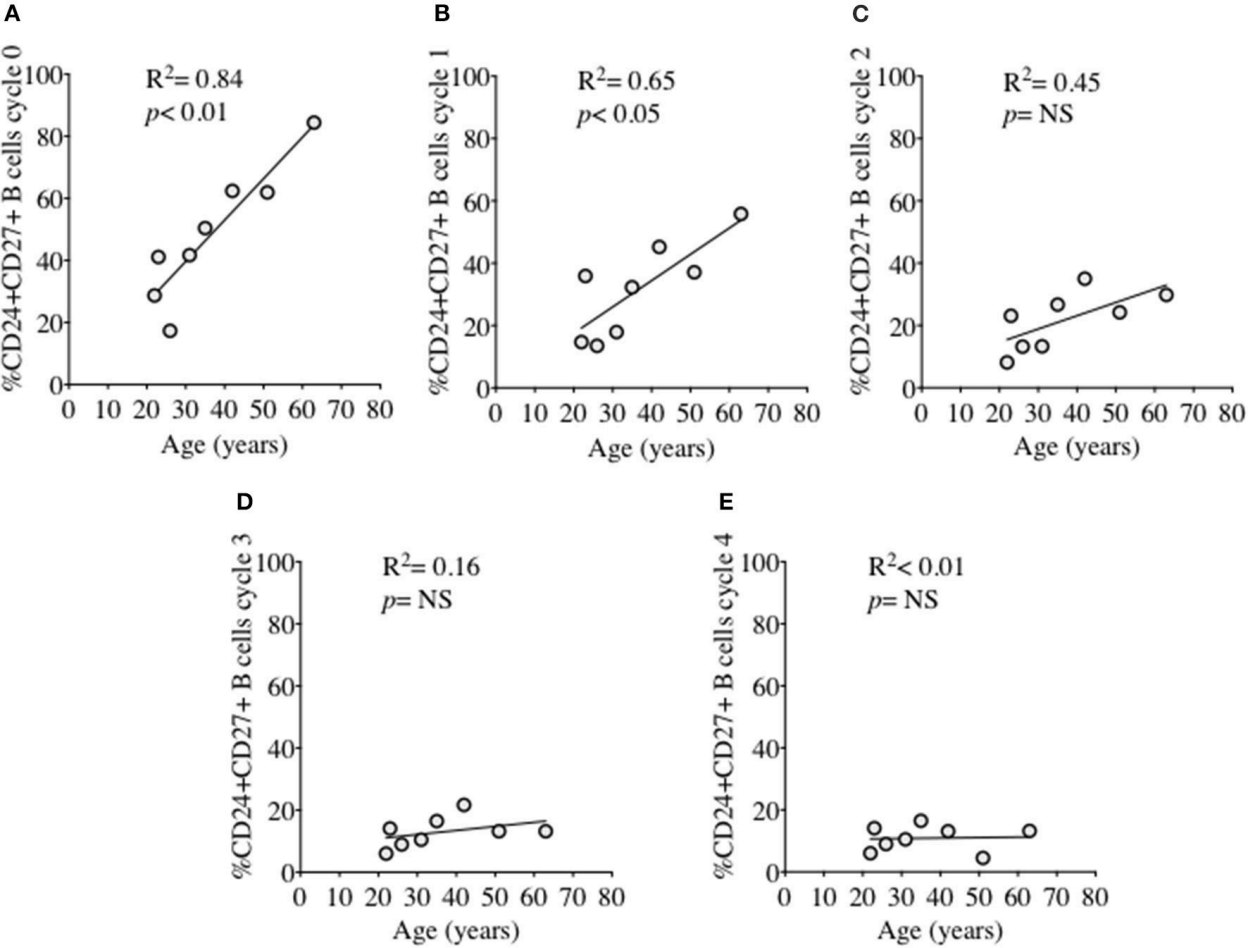
Relationship between %CD24+CD27+ B cells and age over sequential cycles of proliferation. %CD24+CD27+ B cells in cycle 0–4 **(A–E)** in relationship with age (years) after CpG + (anti-IgM+IL2) stimulation scatter plot with regression line is shown (HC; *N* = 8). Pearson correlation (*R*^2^) for Linear regression and *p*-values are shown. NS, not significant at 5% level.

In order to investigate the effect of proliferation on %CD24+ B cells, negatively isolated B cells from freshly isolated PBMCs were stained with CFSE and cultured for 5 days in the presence of CpG + (anti-IgM+IL2) stimulation. CpG stimulation is known to induce multiple proliferation cycles of human B cells (25). We confirmed that stimulation with CpG induced more proliferation cycles and significantly higher IgM and IgG antibody production over 5 days compared to anti-CD40 (TD) stimulation (Supplementary Figure 2) and therefore results for CpG stimulation are shown. After culture B cells were stained with a viability stain and fluorescent conjugates against CD19, CD27, and CD24. Figure 3A shows a representative plot of sequential cycles during B cell proliferation of healthy controls after a 5-day culture with CpG stimulation. The frequency of B cells (Figure 3B) and CD24+ B cells (Figure 3C) within CD27− and CD27+ subsets are shown over each cycle. Both B cell subsets proliferated in response to CpG stimulus, but the percentage of CD24+ B cells decreased with each proliferation cycle in both CD27− and CD27+ B cells. Although there was a higher relative frequency of %CD24+ B cells in CD27+ vs. CD27− B cells at baseline, the rate of decrease was similar in both subsets. Anti-CD40 (TD) stimulated B cells showed similar patterns as CpG stimulation, although with fewer proliferation cycles reached within 5 days culture (data not shown and Supplementary Figure 2). When the percentage of CD24+CD27+ B cells within each cycle was analyzed in relation to baseline parameters in healthy controls, there was a significant positive correlation between age and %CD24+CD27+ B cells in Cycle 0, which was lost over sequential proliferation cycles (Figure 4).

### Changes in mitochondrial mass over sequential proliferation cycles following TI stimulation in naïve and memory B cells

**Figure 5:**
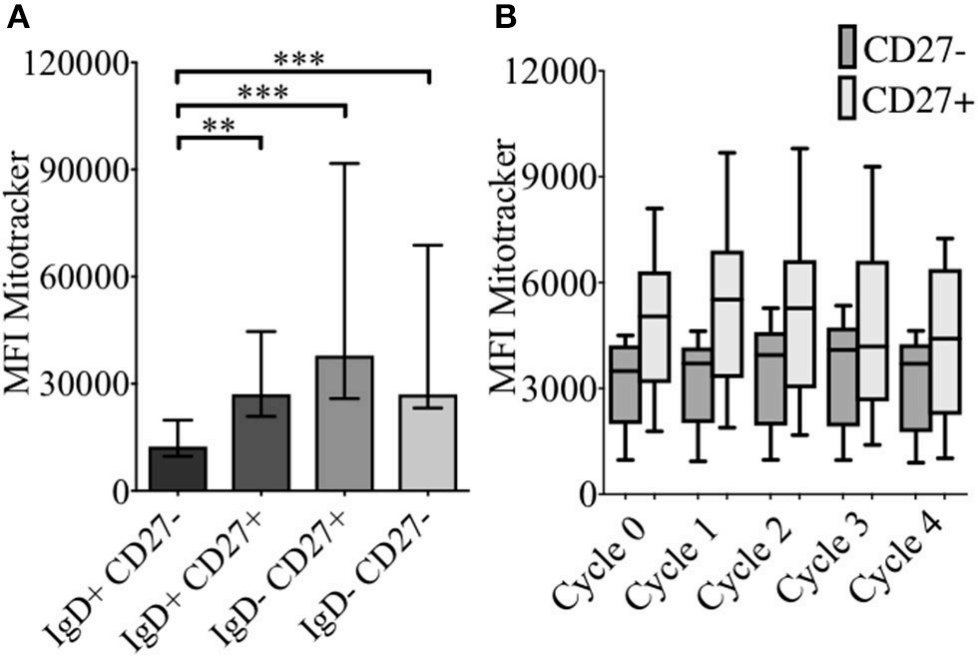
Baseline levels and effect of CpG + (anti-IgM+IL2) -induced proliferation on mitochondrial mass using MitotrackerTM Red FM (MTR) of B cells from HC. MTR expression (MFI) in baseline B cell subsets (HC; *N* = 11) distinguished using the relative expression of IgD and CD27 is shown **(A)**. Bars show median with interquartile range. ** *p* < 0.01 and *** *p* < 0.001 (Mann- Whitney test). MTR expression in CD27− (naïve) and CD27+ (memory) B cells of HC (*N*=8) over sequential cycles of proliferation are shown in box and whiskers plots with median and 10th to 90th percentile in **(B)**.

As energy demand changes during B cell differentiation, we measured mitochondrial mass (MM) in relation to CD24 expression on B cell subsets and cultured B cells with Mitotracker^TM^ Red FM (MTR). MTR is a fluorescent dye which stains mitochondria within live cells, where its accumulation is dependent upon intact cell membrane potential. In Figure 5A MM of B cells from freshly isolated PBMCs, defined by IgD/CD27, are shown. Compared to naïve B cells (IgD+CD27−), memory B cells showed increased MM, with class-switched memory B cells (IgD-CD27+), pre-switched memory B cells (IgD+CD27+), which predominantly secrete IgM and late resting or double negative memory cells (26) (IgD-CD27−) also showing a significant increase in MM.

**Figure 6:**
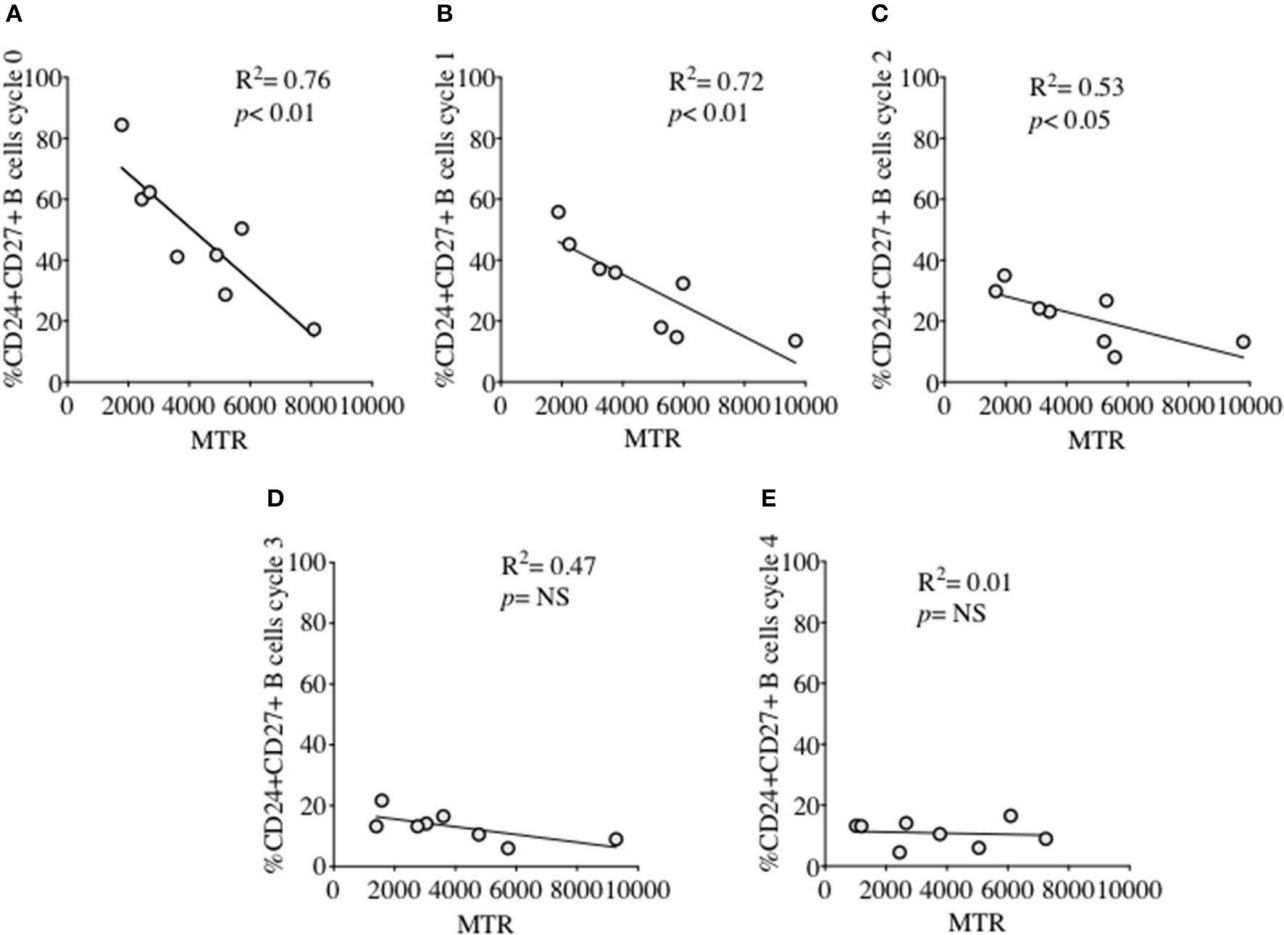
Relationship between %CD24+CD27+ B cells and Mitochondrial mass over sequential cycles of proliferation. %CD24+CD27+ B cells in cycle 0–4 **(A–E)** in relation to Mitochondrial mass (MTR) after CpG + (anti-IgM+IL2) stimulation scatter plot with regression line is shown (HC; *N* = 8). Pearson correlation (*R*^2^) for Linear regression and *p*-values are shown. NS, not significant at 5% level.

MM was also measured over each proliferation cycle in naïve and memory B cells from HC after 5 days of culture. As shown in Figure 5B there was no difference in MM between each proliferation cycle, although the higher MM of memory B cells compared with naïve B cells was maintained over sequential cycles. The correlation between %CD24+CD27+ memory B cells and MM over sequential proliferation cycles after CpG stimulation in HC was also investigated within individual proliferation cycles (Figure 6). CD24+CD27+ B cells in Cycle 0 (Figure 6A) had a strong negative correlation with mitochondrial mass. Over subsequent cycles, this correlation was gradually lost with proliferation and increased percentages of CD24- compared with CD24+memory B cells (Figures 6B–E).

### Phosphorylated AMPK and CD24 in naïve and memory B cells

**Figure 7:**
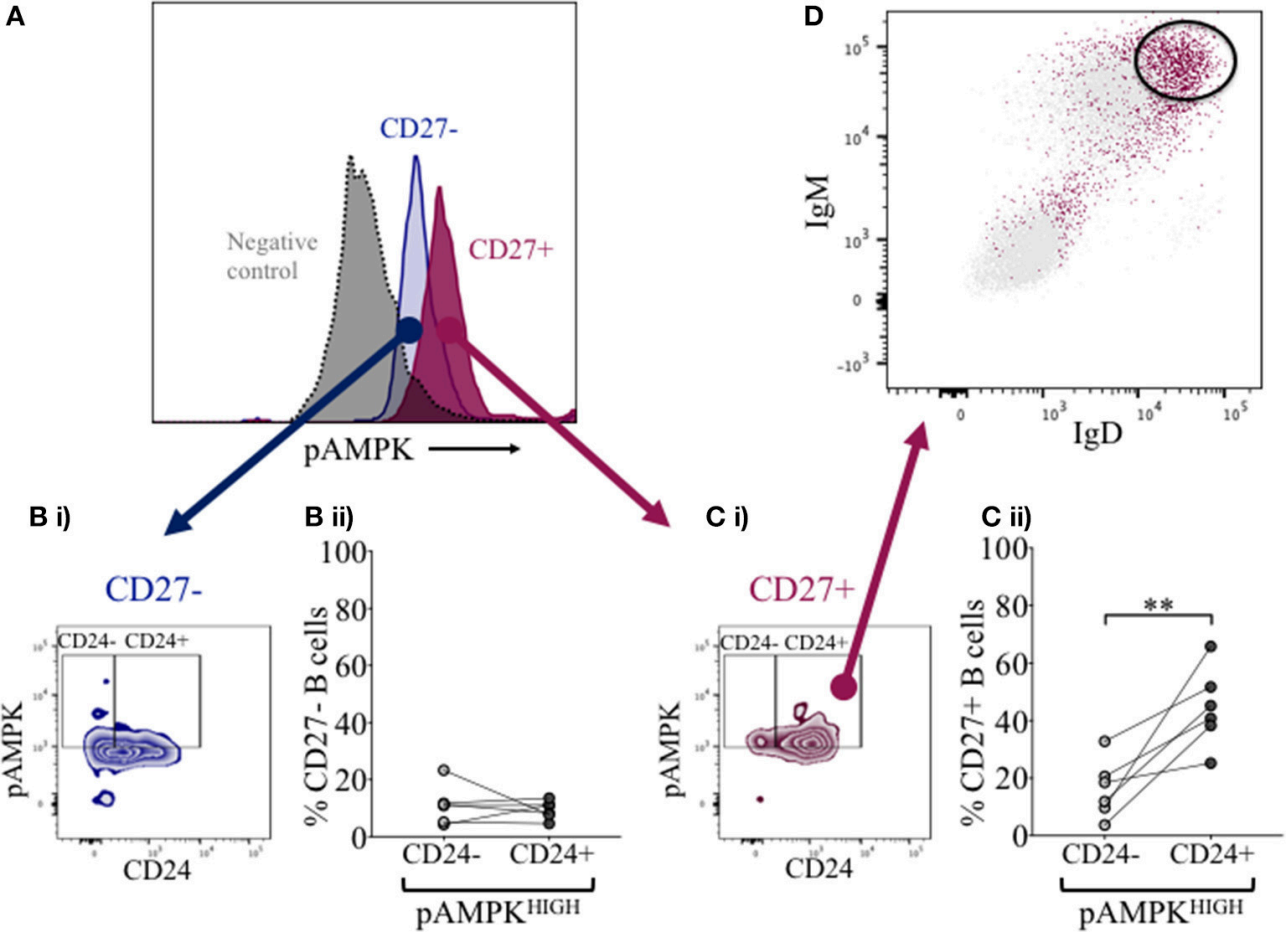
Expression of pAMPK and CD24 in CD27− and CD27+ B cells. Flow cytometric histogram shows a representative plot of pAMPK expression in CD27− (blue) and CD27+ (red) B cells and a negative control without secondary antibody (gray; **A)**. CD24 expression in relation to pAMPK in naïve **(Bi)** and memory **(Ci)** B cell subsets are shown in a contour plot. Percentage B cells in CD24+ (dark gray symbols) and CD24- B cells (light gray symbols) populations positive for pAMPK in healthy controls for CD27− **(Bii)** and CD27+ **(Cii)** B cells are shown for HC (*N* = 6). Flow cytometric dot plot shows how IgD and IgM is expressed on CD24+ pAMPKHIGH CD27+ memory B cells (red) vs. the rest of the population in CD27+ memory B cells **(D)**. **p <0.01; Wilcoxon signed rank test.

Overexpression of CD24 has been described on a number of human tumor cells, including acute myeloid leukemia (27–29). Microarray analyses of prostate cancer cells suggested that CD24 had a role as a growth-promoting factor, which was downregulated when the enzyme adenosine monophosphate kinase (AMPK) was inactivated (30). The kinase is activated (phosphorylated -pAMPK) in response to stresses that deplete cellular ATP supplies such as low glucose, hypoxia and exercise positively regulates signaling pathways that replenish ATP, including fatty acid oxidation, autophagy and mitochondrial biogenesis. In normal human B cells, it has been reported that only memory B cells, and especially late memory B cells (IgD-CD27−), express senescence-associated secretory phenotypes (SASP), which are associated with spontaneous activation of AMPK (31). We therefore examined the potential relationship between CD24 on B cells, separated on the basis of CD27 expression, and phosphorylation of AMPK, using Phospho-Flow. In Figure 7A a representative plot of pAMPK expression on CD27− and memory CD27+ B cells in comparison with a negative control (pAMPK stain without secondary antibody) are shown. The median expression (MFI) of pAMPK on CD27−B cells were used as a cut-off for high pAMPK expression (referred to as pAMPK-HIGH) in comparison with memory B cells (Figures 7Bi, 7Ci). Approximately 20% of naïve B cells showed a “pAMPK-HIGH” expression, which was equally distributed between CD24- and CD24+ B cells (Figure 7Bii). In CD27+ memory B cells pAMPK-HIGH expression was shown to be at a much higher frequency than in naïve B cells, particularly in the CD24+ population. Thus, this indicated a clear-cut difference in AMPK signaling in memory B cells compared to CD27−B cells, which was significant for CD24+ compared with CD24- memory B cells (Figure 7Cii).

### Identity of pampk-high population with CD24+CD27+ memory B cells

Additional surface markers were then included in order to identify the B cell subpopulation associated with pAMPK-HIGH CD24+CD27+ memory B cells more precisely. As shown in Figure 7D, CD24+CD27+ memory B cells in the pAMPK-HIGH group were predominantly within the IgD++IgM++ (MFI) population. The remainder of CD24+CD27+ memory B cells which were not pAMPK-HIGH, were mostly IgD-IgM- (switched memory B cell phenotype) although there was also a significant population of IgD++IgM++ B cells. The memory (CD27+) B cell subset co-expressing IgD and IgM represent a marginal zone or pre-switch memory B cell phenotype (IgD+CD38-).

### B cells expressing CD24 and other cell surface molecules in relation to glucose consumption and lactate production

**Figure 8:**
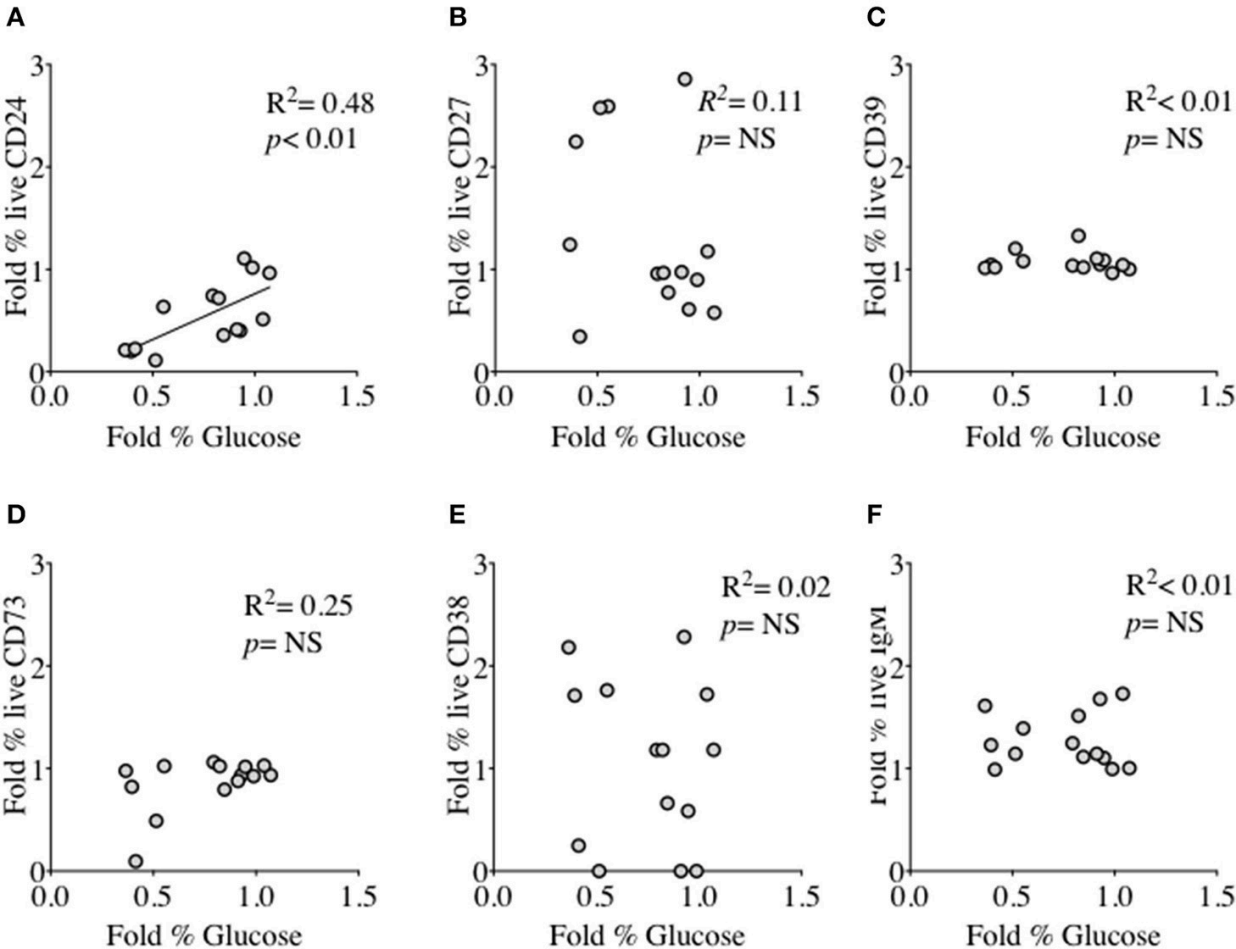
Relationship between Fold % changes in Glucose concentration and B cell membrane molecules following *in vitro* culture in the presence of anti-CD40 + (anti-IgM+Il2) an CpG + (anti-IgM+IL2). Fold % changes in Glucose measured in the supernatant of B cell cultures vs. the expression of CD24 **(A)**, CD27 **(B)**, CD39 **(C)**, CD73 **(D)**, CD38 **(E)** and IgM **(F)** in live B cells are shown. Pearson correlation (*R*^2^) for Linear regression and exact *p*-values are shown (*N* = 6). NS, not significant at 5% level.

**Figure 9:**
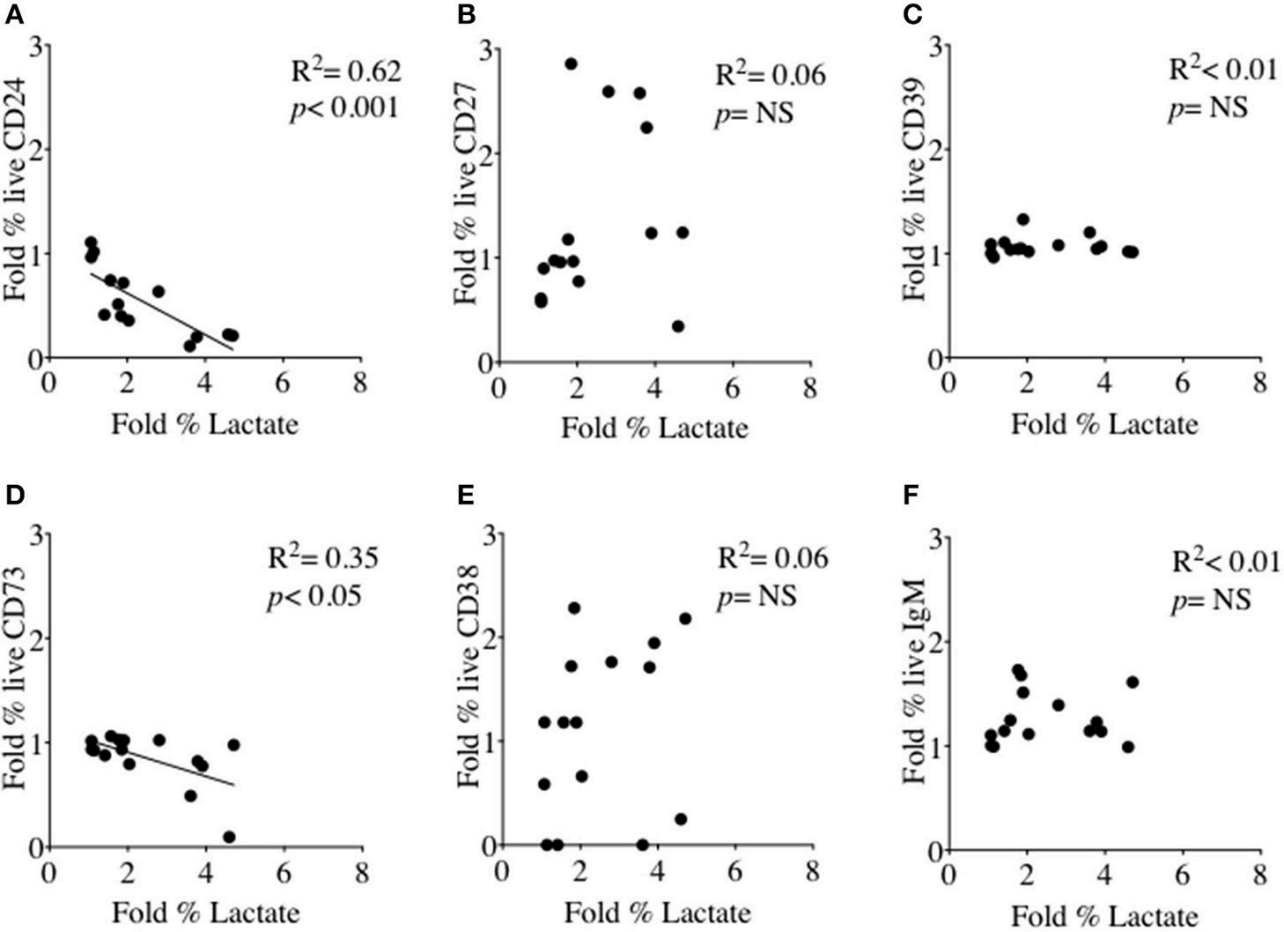
Relationship between Fold % changes in Lactate concentration and B cell membrane molecules following *in vitro* culture in the presence of anti-CD40 + (anti-IgM+Il2) an CpG + (anti-IgM+IL2). Fold % changes in Lactate measured in the supernatant of B cell cultures vs. the expression of CD24 **(A)**, CD27 **(B)**, CD39 **(C)**, CD73 **(D)**, CD38 **(E)** and IgM **(F)** in live B cells are shown. Pearson correlation (*R*^2^) for Linear regression and exact *p*-values are shown (*N* = 6). NS, not significant at 5% level.

We had thus shown a tentative association between CD24 expression and energy metabolism in the form of phosphorylation of AMPK. We extended our analysis to cultured B cells to explore the relationship between a direct measure of 2 key metabolites namely glucose (substrate) and lactate (product) during *in vitro* stimulation of B cells as a proxy for glycolysis, and B cell immunophenotype markers including CD24 and those associated with ATP metabolism. Additional markers were the Ectonucleotidases CD39 and CD73 and cyclic ADP Ribose hydrolase (CD38) and CD27 (a differentiation marker expressed on most Memory B cells) and the B cell receptor, IgM. Figure 8 (glucose) and Figure 9 (lactate) show fold changes in percentages of CD24 **(A)**, CD27 **(B)**, CD39 **(C)**, CD73 **(D)**, CD38 **(E)**, and IgM **(F)** in relation to fold changes in proportion of glucose and lactate between days 1 and 3. We found that the direction of fold changes in both %glucose (negative) and %lactate (positive) in culture supernatants correlated with %live CD24+ B cells (*p* < 0.01 and *p* < 0.001, respectively) but not with those of other phenotypic markers, except a weaker (positive) association between CD73 and lactate production (*p* < 0.05).

### Effect of B cell receptor ligation on cell viability and CD24 expression on CD27− and CD27+ B cells

**Figure 10:**
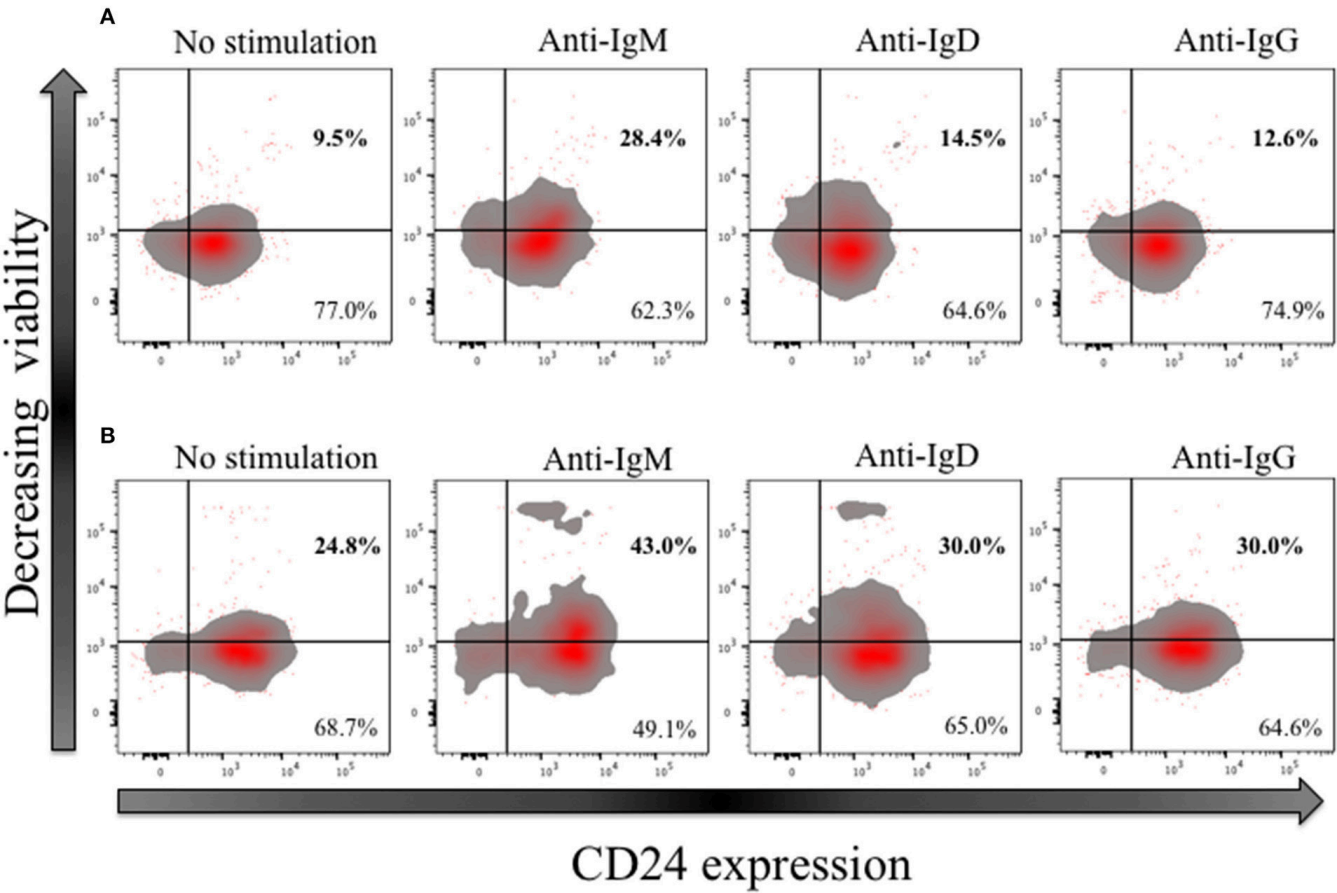
Effect of short-term (3 h) crosslinking of the B cell receptor on the viability of CD24+ B cells. Representative Flow cytometric density plots with outliers of CD27− **(A)** and CD27+ **(B)** B cells of HC in the absence and presence of BCR stimulation with anti-IgM, anti-IgD and anti-IgG cultured for 3 h. Plots show the expression of CD24; CD24- and CD24+ B cells on the x-axis and viability stain on the y-axis.

In early naïve pro-B cells, CD24 co-localizes with and modifies the function of other receptors, most notably the B cell receptor (BCR) (32), but its role in the normal B cell development outside the bone marrow has not been extensively explored in man. We and others have earlier showed that CD24 has a higher expression (MFI) in memory compared to mature naïve B cells. We thus tested the effect of BCR stimulation with anti-IgM, anti-IgD and anti-IgG antibodies (Goat anti-human polyclonal antibodies) on B cell viability in CD27− (naive) and CD27+ (memory) CD24+ B cells. Freshly isolated B cells from healthy donors were cultured for 3 h at 37°C in the absence or presence of isotype-specific BCR cross-linking. After incubation, B cells were harvested and stained for viability, and with fluorescent conjugates against CD19, CD27, and CD24. Figure 10 shows a representative plot of B cells divided on the basis of CD24 expression in naïve (Figure 10A) and memory B cells (Figure 10B). Within the CD24+ B cells, subpopulations were identified in relation to decreasing viability. After cross linking the BCR in both naïve and memory B cells, there was a marked decrease in viable B cells cultured with anti-IgM. The percentage of viable B cells was significantly less in both naïve and memory B cells anti-IgM incubated cultures compared with those incubated with anti-IgD or anti-IgG (data not shown; *p* < *0.05* for all comparisons). This may reflect the higher number (as shown by MFI) of IgM receptors present on the surface of B cells, compared to IgD and IgG. Nevertheless, following notably IgM cross-linking CD24+ B cells were prominent in having reducing viability.

## Discussion

Although CD24 plays a well-described role in early B cell development in the bone marrow in mice and man, the role of an increased frequency and expression of CD24 in human memory B cells compared with mature naïve B cells has not been thoroughly explored. Our novel finding of increased CD24 expression and frequency within IgD+ memory B cell populations, as well as IgD+ naïve B cells in ME/CFS patients compared with healthy controls therefore prompted the investigation of CD24 throughout B cell maturation. CD24 is a cell adhesion molecule known to mediate signal transduction including intracellular calcium mobilization and phosphorylation of intracellular proteins (33–35). We found that CD24 has a different role within naïve and memory B cell populations related to AMPK phosphorylation, and which was mainly confined to un-switched memory B cells with dual IgD and IgM surface expression.

We used *in vitro* culture and stimulation of isolated B cells to follow—CD24 positivity during differentiation. In the absence of stimulation, an increased frequency of viable CD24+ B cells was found in cultures from ME/CFS patients compared to healthy controls. This *in vitro* finding was in line with what we have reported in our previous *ex vivo* whole blood phenotype study (16). Over sequential cycles of proliferation in response to T-independent stimulation of B cells from healthy donors, there was an incremental decrease in frequency of CD24+ B cells in both naïve and memory B cells, as defined by CD27, in parallel with naïve B cell differentiation. CD27 is a marker for memory B cells, both unswitched (IgD+CD27+) and switched (IgD-CD27+). However, a small population of memory B cells which has lost CD27, has been identified and is thought to represent a late or exhausted memory B cell sub-population (26, 36). Interestingly, CD24 retention after stimulation in non-proliferating (cycle 0) memory B cells was related to age in healthy controls, suggesting that continued expression of CD24 might act as an unresponsiveness/senescence-associated marker on memory B cells.

CD24 expression on human B cells has been largely utilized as an immune-phenotype marker for early stage B cells where it is highest on newly exited (from bone marrow) transitional B cells. Expression then sharply decreases in mature naïve B cells. We confirmed a differentiation-dependent expression (MFI) of CD24 which was significantly higher in memory B cells compared to mature naïve B cells. As a cell-adhesion molecule, CD24 can ligate different specific signaling partners on target cells in a B cell subset dependent manner. The ability of CD24 to associate with other cell surface receptors such as L-Selectin, L-1 cell adhesion molecules (L1CAM) and Siglec-G also has consequences for downstream signaling‘ potential (37–39). For example, its synergism with the BCR in normal human B cells was demonstrated in relation to apoptosis (8). These findings prompted us to investigate how BCR engagement affects CD24+ B cell viability in naïve compared with memory B cell subsets from peripheral blood. We tested this by short-term cultures with isotype specific BCR engagement. A decrease in viability of CD24+ B cells was found to be most marked following ligation with anti-IgM compared with anti-IgD or anti-IgG in both naïve and memory B cell populations, suggesting a possible preferential co-localization with IgM-class BCR.

Signaling pathways triggered following antigen and co-stimulus encounter in B cells regulate cell cycle entry, effector function and also the type of metabolic pathways used for energy production. Naïve B cells, post-GC memory B cells and plasma cells have different metabolic

Requirements and it is also likely that their responses to metabolic stress may differ at different stages of differentiation. Naïve B cells for example, depend largely on oxidative phosphorylation associated with metabolites of the TCA cycle rather than those of glycolysis, whereas post germinal center B cells, as we confirmed in human peripheral blood memory B cells, have both increased glucose consumption and mitochondrial mass (40). In activated B cells (and germinal center reactions) it is thus a balance between allowing rapid proliferation and differentiation whilst managing the generation of damaging reactive oxygen species, and, *in vivo*, perhaps limitations of nutrient provision. We showed a clear difference between naïve and memory B cells with respect to CD24 and energy metabolism, namely phosphorylation of AMPK in CD24+ and not CD24 negative memory B cells from peripheral blood. There was no such association with phosphorylation of AMPK and CD24 in naïve B cells. It is possible that the use of pro-catabolic pathways of energy production (such as AMPK) in response to cell stress may be necessary to aid cell survival at this stage, with a resultant downstream triggering of autophagy and other regulators of cellular metabolism to limit excessive activation. CD24 could thus be involved in different downstream metabolic actions associated with AMPK. Further phenotype analysis showed that pAMPK-HIGH and CD24+ memory B cells were present in the CD27+ subset expressing high IgD and IgM surface markers. This confirmed earlier findings by Sanz et al describing a link between CD24 and un-switched memory B cells (15). Phosphorylation of AMPK has also been shown to be one of the signals throughout metabolic pathways which is associated with senescence and low/absent responsiveness in memory B cells (31). As senescent B cells increase with age this may be reflected by the increased retention of CD24 on memory B cells which failed to proliferate following *in vitro* stimulation and which we also found to have a positive correlation with age in healthy individuals. Mitochondrial mass was also found to be lowest in CD24+ B cells remaining in cycle 0 after *in vitro* stimulation, which may also indicate usage of a preferred energy source for these possibly senescent B cells, and possibly in B cells from ME/CFS patients with higher CD24 expression. There was also confirmed by the finding of a strong negative correlation between CD24 positivity and surrogate measures of glycolysis (glucose/lactate ratios).

Abnormalities in energy pathway usage has already been described as an important feature in understanding the pathophysiology and etiology in ME/CFS (21, 41). The metabolic anomalies described in sera from patients with ME/CFS have been proposed to be regulated by increased AMPK phosphorylation (42). This would thus appear to be confirmed by the finding of increased expression of CD24 on B cells from ME/CFS patients and its relationship to AMPK and surrogate measures of glycolysis, namely glucose consumption and lactate production as observed in this study. Increased expression of CD24 which we have described on IgD+ B cells in peripheral blood of ME/CFS patients could thus reflect abnormalities in maintaining appropriate ATP generation perhaps mediated through inappropriate activation of AMPK.

## Conclusion

Previous studies have focused on the engagement of CD24 early in B cell development and when expressed in transformed cancer cells, where it plays an important role as an adhesion molecule regulating survival and allowing spread throughout tissues and lymphatics respectively. We investigated the relationship between CD24 expression and B cell differentiation. In memory B cells, we found that CD24 seems to have another role as shown by an association with phosphorylation of AMPK particularly in B cells co-expressing high IgM and IgD surface markers, but not in naïve or switched memory B cells. According to our *in vitro* findings, it is more likely that CD24-negative B cells had undergone productive proliferation, and that CD24+ B cells were more prone to unresponsiveness or cell death upon stimulation and/or BCR engagement alone. We therefore conclude that CD24 expression on B cells is related to energy metabolism throughout differentiation and that its role differs between B cell subsets.

## Ethics statement

This study was carried out in accordance with the recommendations of a diagnosis of ME/CFS in patients fulfilling consensus criteria (Canadian, CDC and Fukuda). All subjects (patients and healthy controls) gave written informed consent in accordance with the Declaration of Helsinki. The protocol was approved by the NRES Committee London–City Road and Hampstead Research ethics Committee (REC reference: 14/LO/0388).Patients were selected for the study by SB (Royal London Hospital of Integrated Medicine) and AB (St Helier Hospital NHS Trust). Clinical assessments were then performed to assess patients' eligibility for study inclusion. Patients were informed verbally about the study and additionally given information sheets with written informed consent.

## Author contributions

FM collected samples, developed techniques, and performed all the experiments under the supervision of GC and in collaboration with CA. FM and GC wrote the manuscript. ML and VR contributed throughout to discussion and interpretation of results, together with CA. AB, SB, ML, and VR reviewed, commented on and had their suggested changes incorporated into the manuscript. Clinical assessments and recruitment of patients was by AB and SB. All authors also had access to raw data.

### Conflict of interest statement

The authors declare that the research was conducted in the absence of any commercial or financial relationships that could be construed as a potential conflict of interest.
