# Supplementary figures and images for: CD24 Expression and B Cell Maturation Shows a Novel Link With Energy Metabolism: Potential Implications for Patients With Myalgic Encephalomyelitis/Chronic Fatigue Syndrome
Source: Front Immunol. 2018 Oct 22;9:2421. doi: 10.3389/fimmu.2018.02421 (PMC6204382; doi:10.3389/fimmu.2018.02421)

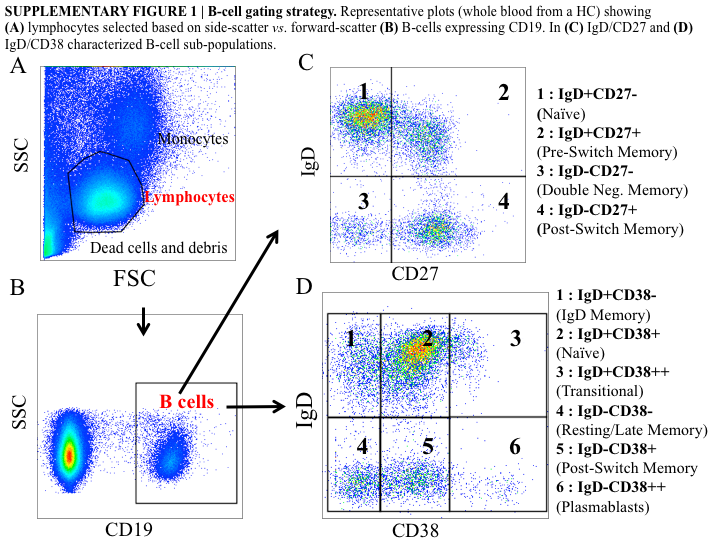

Supplement: Supplementary file 1 [file Image_1.TIFF]

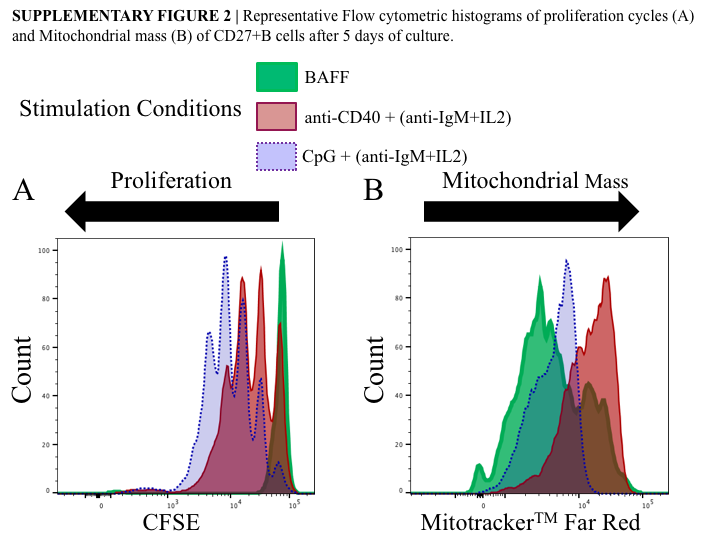

Supplement: Supplementary file 2 [file Image_2.TIFF]
